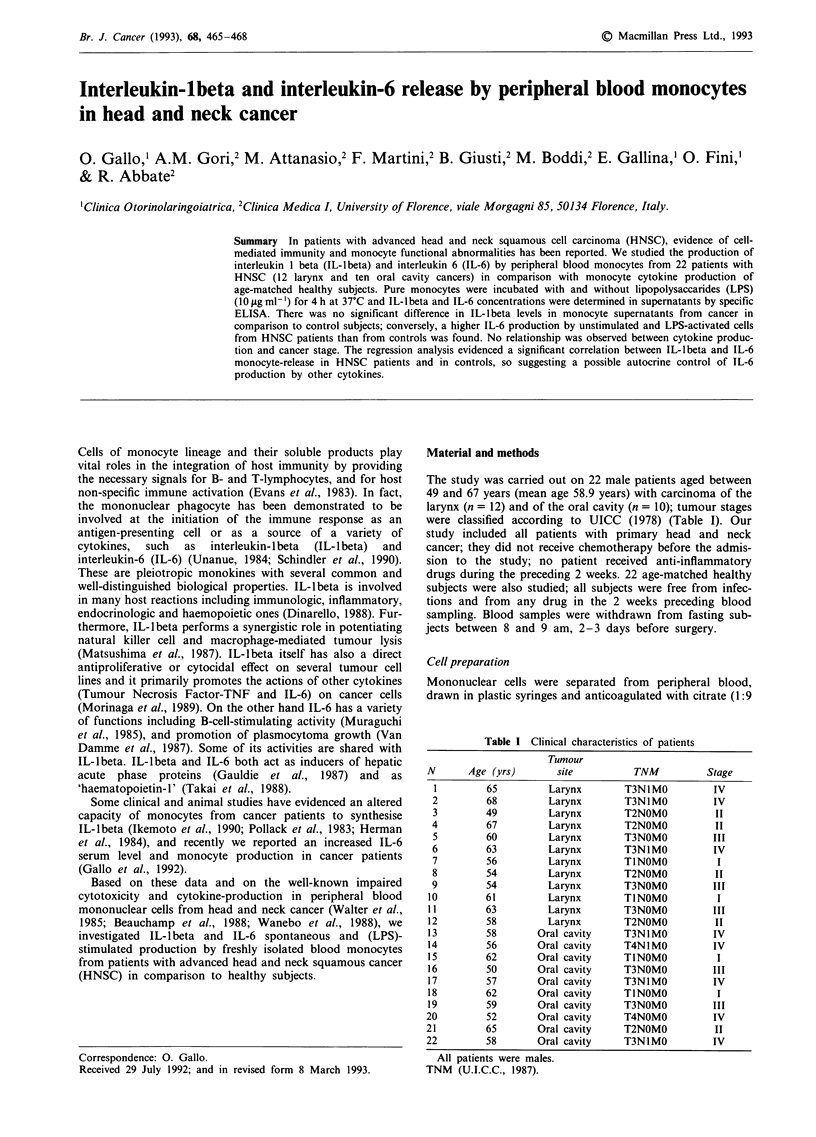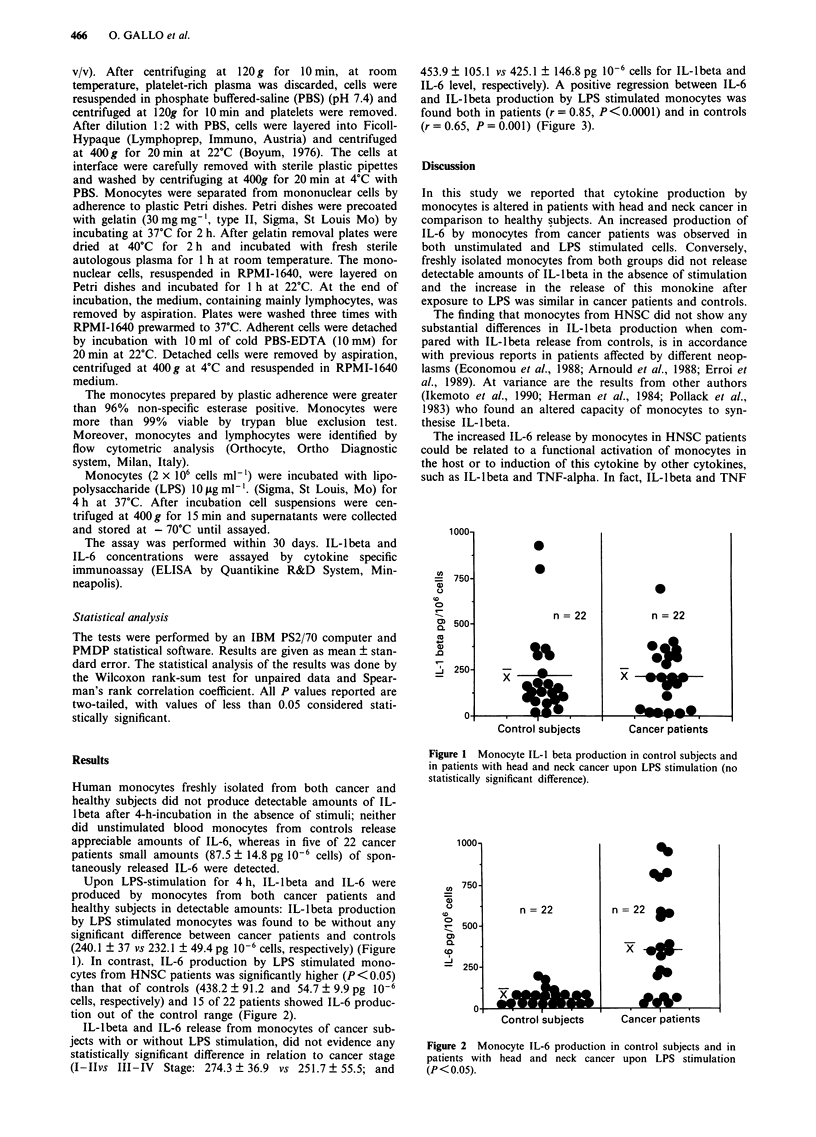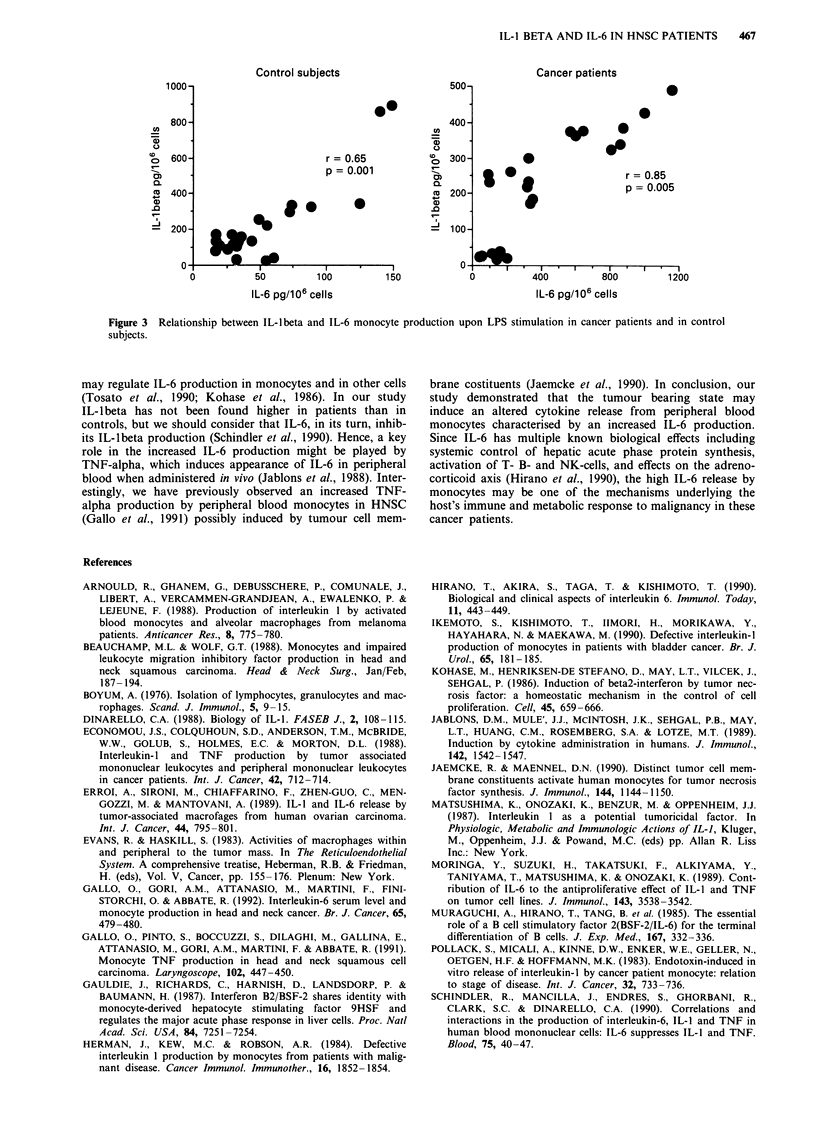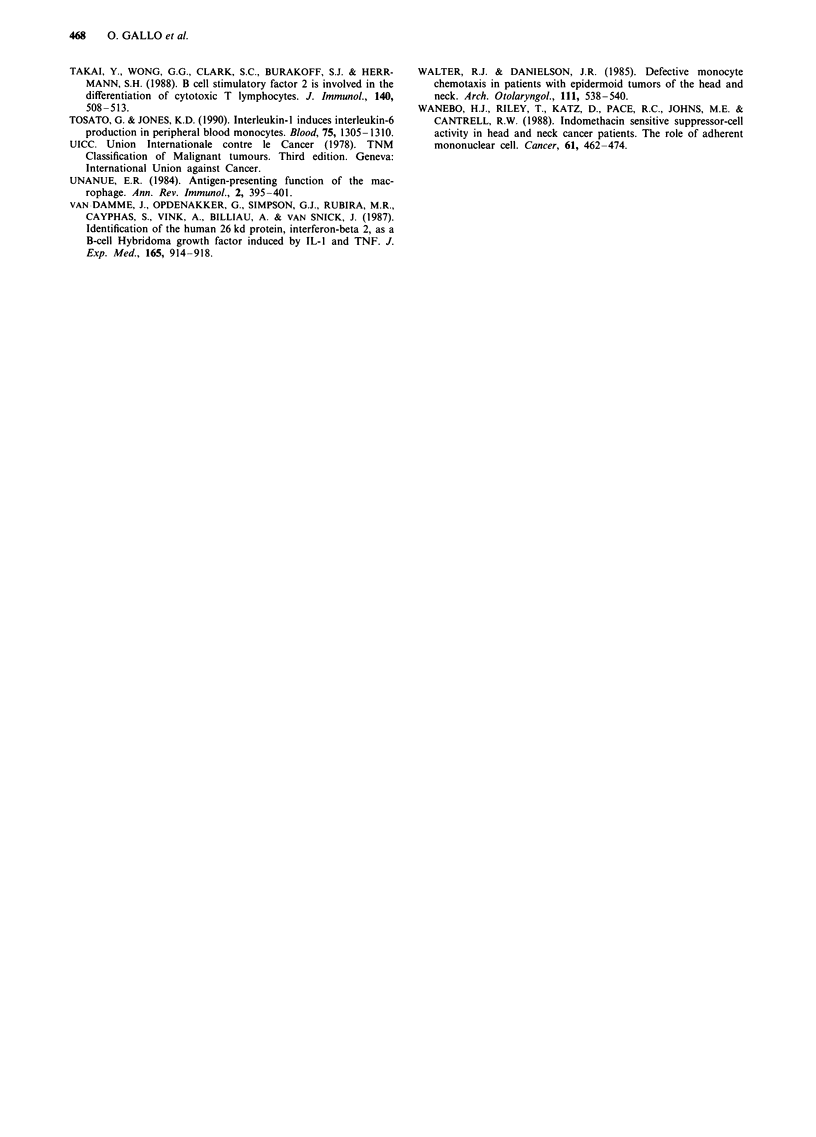# Interleukin-1 beta and interleukin-6 release by peripheral blood monocytes in head and neck cancer.

**DOI:** 10.1038/bjc.1993.371

**Published:** 1993-09

**Authors:** O. Gallo, A. M. Gori, M. Attanasio, F. Martini, B. Giusti, M. Boddi, E. Gallina, O. Fini, R. Abbate

**Affiliations:** Clinica Otorinolaringoiatrica, University of Florence, Italy.

## Abstract

In patients with advanced head and neck squamous cell carcinoma (HNSC), evidence of cell-mediated immunity and monocyte functional abnormalities has been reported. We studied the production of interleukin 1 beta (IL-1 beta) and interleukin 6 (IL-6) by peripheral blood monocytes from 22 patients with HNSC (12 larynx and ten oral cavity cancers) in comparison with monocyte cytokine production of age-matched healthy subjects. Pure monocytes were incubated with and without lipopolysaccharides (LPS) (10 micrograms ml-1) for 4 h at 37 degrees C and IL-1 beta and IL-6 concentrations were determined in supernatants by specific ELISA. There was no significant difference in IL-1 beta levels in monocyte supernatants from cancer in comparison to control subjects; conversely, a higher IL-6 production by unstimulated and LPS-activated cells from HNSC patients than from controls was found. No relationship was observed between cytokine production and cancer stage. The regression analysis evidenced a significant correlation between IL-1 beta and IL-6 monocyte-release in HNSC patients and in controls, so suggesting a possible autocrine control of IL-6 production by other cytokines.


Br. J. Cancer (1993), 68, 465–468 © Macmillan Press Ltd., 1993

### Interleukin-1beta and interleukin-6 release by peripheral blood monocytes in head and neck cancer

O. Gallo,[1] A.M. Gori,[2] M. Attanasio,[2] F. Martini,[2] B. Giusti,[2] M. Boddi,[2] E. Gallina,[1] O. Fini,[1] & R. Abbate[2]

[1]Clinica Otorinolaringoiatrica, [2]Clinica Medica I, University of Florence, viale Morgagni 85, 50134 Florence, Italy.

**Summary** In patients with advanced head and neck squamous cell carcinoma (HNSC), evidence of cell-mediated immunity and monocyte functional abnormalities has been reported. We studied the production of interleukin 1 beta (IL-1beta) and interleukin 6 (IL-6) by peripheral blood monocytes from 22 patients with HNSC (12 larynx and ten oral cavity cancers) in comparison with monocyte cytokine production of age-matched healthy subjects. Pure monocytes were incubated with and without lipopolysaccarides (LPS) ($10\,\mu g\,ml^{-1}$) for 4 h at 37°C and IL-1beta and IL-6 concentrations were determined in supernatants by specific ELISA. There was no significant difference in IL-1beta levels in monocyte supernatants from cancer in comparison to control subjects; conversely, a higher IL-6 production by unstimulated and LPS-activated cells from HNSC patients than from controls was found. No relationship was observed between cytokine production and cancer stage. The regression analysis evidenced a significant correlation between IL-1beta and IL-6 monocyte-release in HNSC patients and in controls, so suggesting a possible autocrine control of IL-6 production by other cytokines.

Cells of monocyte lineage and their soluble products play vital roles in the integration of host immunity by providing the necessary signals for B- and T-lymphocytes, and for host non-specific immune activation (Evans *et al.*, 1983). In fact, the mononuclear phagocyte has been demonstrated to be involved at the initiation of the immune response as an antigen-presenting cell or as a source of a variety of cytokines, such as interleukin-1beta (IL-1beta) and interleukin-6 (IL-6) (Unanue, 1984; Schindler *et al.*, 1990). These are pleiotropic monokines with several common and well-distinguished biological properties. IL-1beta is involved in many host reactions including immunologic, inflammatory, endocrinologic and haemopoietic ones (Dinarello, 1988). Furthermore, IL-1beta performs a synergistic role in potentiating natural killer cell and macrophage-mediated tumour lysis (Matsushima *et al.*, 1987). IL-1beta itself has also a direct antiproliferative or cytocidal effect on several tumour cell lines and it primarily promotes the actions of other cytokines (Tumour Necrosis Factor-TNF and IL-6) on cancer cells (Morinaga *et al.*, 1989). On the other hand IL-6 has a variety of functions including B-cell-stimulating activity (Muraguchi *et al.*, 1985), and promotion of plasmocytoma growth (Van Damme *et al.*, 1987). Some of its activities are shared with IL-1beta. IL-1beta and IL-6 both act as inducers of hepatic acute phase proteins (Gauldie *et al.*, 1987) and as 'haematopoietin-1' (Takai *et al.*, 1988).

Some clinical and animal studies have evidenced an altered capacity of monocytes from cancer patients to synthesise IL-1beta (Ikemoto *et al.*, 1990; Pollack *et al.*, 1983; Herman *et al.*, 1984), and recently we reported an increased IL-6 serum level and monocyte production in cancer patients (Gallo *et al.*, 1992).

Based on these data and on the well-known impaired cytotoxicity and cytokine-production in peripheral blood mononuclear cells from head and neck cancer (Walter *et al.*, 1985; Beauchamp *et al.*, 1988; Wanebo *et al.*, 1988), we investigated IL-1beta and IL-6 spontaneous and (LPS)-stimulated production by freshly isolated blood monocytes from patients with advanced head and neck squamous cancer (HNSC) in comparison to healthy subjects.

Correspondence: O. Gallo.
Received 29 July 1992; and in revised form 8 March 1993.

### Material and methods

The study was carried out on 22 male patients aged between 49 and 67 years (mean age 58.9 years) with carcinoma of the larynx ($n = 12$) and of the oral cavity ($n = 10$); tumour stages were classified according to UICC (1978) (Table I). Our study included all patients with primary head and neck cancer; they did not receive chemotherapy before the admission to the study; no patient received anti-inflammatory drugs during the preceding 2 weeks. 22 age-matched healthy subjects were also studied; all subjects were free from infections and from any drug in the 2 weeks preceding blood sampling. Blood samples were withdrawn from fasting subjects between 8 and 9 am, 2–3 days before surgery.

### *Cell preparation*

Mononuclear cells were separated from peripheral blood, drawn in plastic syringes and anticoagulated with citrate (1:9

**Table I** Clinical characteristics of patients

| *N* | *Age (yrs)* | *Tumour site* | *TNM* | *Stage* |
|---|---|---|---|---|
| 1 | 65 | Larynx | T3N1M0 | IV |
| 2 | 68 | Larynx | T3N1M0 | IV |
| 3 | 49 | Larynx | T2N0M0 | II |
| 4 | 67 | Larynx | T2N0M0 | II |
| 5 | 60 | Larynx | T3N0M0 | III |
| 6 | 63 | Larynx | T3N1M0 | IV |
| 7 | 56 | Larynx | T1N0M0 | I |
| 8 | 54 | Larynx | T2N0M0 | II |
| 9 | 54 | Larynx | T3N0M0 | III |
| 10 | 61 | Larynx | T1N0M0 | I |
| 11 | 63 | Larynx | T3N0M0 | III |
| 12 | 58 | Larynx | T2N0M0 | II |
| 13 | 58 | Oral cavity | T3N1M0 | IV |
| 14 | 56 | Oral cavity | T4N1M0 | IV |
| 15 | 62 | Oral cavity | T1N0M0 | I |
| 16 | 50 | Oral cavity | T3N0M0 | III |
| 17 | 57 | Oral cavity | T3N1M0 | IV |
| 18 | 62 | Oral cavity | T1N0M0 | I |
| 19 | 59 | Oral cavity | T3N0M0 | III |
| 20 | 52 | Oral cavity | T4N0M0 | IV |
| 21 | 65 | Oral cavity | T2N0M0 | II |
| 22 | 58 | Oral cavity | T3N1M0 | IV |

All patients were males.
TNM (U.I.C.C., 1987).

v/v). After centrifuging at 120 *g* for 10 min, at room temperature, platelet-rich plasma was discarded, cells were resuspended in phosphate buffered-saline (PBS) (pH 7.4) and centrifuged at 120*g* for 10 min and platelets were removed. After dilution 1:2 with PBS, cells were layered into Ficoll-Hypaque (Lymphoprep, Immuno, Austria) and centrifuged at 400 *g* for 20 min at 22°C (Boyum, 1976). The cells at interface were carefully removed with sterile plastic pipettes and washed by centrifuging at 400*g* for 20 min at 4°C with PBS. Monocytes were separated from mononuclear cells by adherence to plastic Petri dishes. Petri dishes were precoated with gelatin (30 mg $mg^{-1}$, type II, Sigma, St Louis Mo) by incubating at 37°C for 2 h. After gelatin removal plates were dried at 40°C for 2 h and incubated with fresh sterile autologous plasma for 1 h at room temperature. The mononuclear cells, resuspended in RPMI-1640, were layered on Petri dishes and incubated for 1 h at 22°C. At the end of incubation, the medium, containing mainly lymphocytes, was removed by aspiration. Plates were washed three times with RPMI-1640 prewarmed to 37°C. Adherent cells were detached by incubation with 10 ml of cold PBS-EDTA (10 mM) for 20 min at 22°C. Detached cells were removed by aspiration, centrifuged at 400 *g* at 4°C and resuspended in RPMI-1640 medium.

The monocytes prepared by plastic adherence were greater than 96% non-specific esterase positive. Monocytes were more than 99% viable by trypan blue exclusion test. Moreover, monocytes and lymphocytes were identified by flow cytometric analysis (Orthocyte, Ortho Diagnostic system, Milan, Italy).

Monocytes ($2 \times 10^6$ cells $ml^{-1}$) were incubated with lipopolysaccharide (LPS) 10 $\mu g$ $ml^{-1}$. (Sigma, St Louis, Mo) for 4 h at 37°C. After incubation cell suspensions were centrifuged at 400 *g* for 15 min and supernatants were collected and stored at − 70°C until assayed.

The assay was performed within 30 days. IL-1beta and IL-6 concentrations were assayed by cytokine specific immunoassay (ELISA by Quantikine R&D System, Minneapolis).

### *Statistical analysis*

The tests were performed by an IBM PS2/70 computer and PMDP statistical software. Results are given as mean ± standard error. The statistical analysis of the results was done by the Wilcoxon rank-sum test for unpaired data and Spearman's rank correlation coefficient. All *P* values reported are two-tailed, with values of less than 0.05 considered statistically significant.

### Results

Human monocytes freshly isolated from both cancer and healthy subjects did not produce detectable amounts of IL-1beta after 4-h-incubation in the absence of stimuli; neither did unstimulated blood monocytes from controls release appreciable amounts of IL-6, whereas in five of 22 cancer patients small amounts (87.5 ± 14.8 pg $10^{-6}$ cells) of spontaneously released IL-6 were detected.

Upon LPS-stimulation for 4 h, IL-1beta and IL-6 were produced by monocytes from both cancer patients and healthy subjects in detectable amounts: IL-1beta production by LPS stimulated monocytes was found to be without any significant difference between cancer patients and controls (240.1 ± 37 *vs* 232.1 ± 49.4 pg $10^{-6}$ cells, respectively) (Figure 1). In contrast, IL-6 production by LPS stimulated monocytes from HNSC patients was significantly higher ($P < 0.05$) than that of controls (438.2 ± 91.2 and 54.7 ± 9.9 pg $10^{-6}$ cells, respectively) and 15 of 22 patients showed IL-6 production out of the control range (Figure 2).

IL-1beta and IL-6 release from monocytes of cancer subjects with or without LPS stimulation, did not evidence any statistically significant difference in relation to cancer stage (I–II*vs* III–IV Stage: 274.3 ± 36.9 *vs* 251.7 ± 55.5; and 453.9 ± 105.1 *vs* 425.1 ± 146.8 pg $10^{-6}$ cells for IL-1beta and IL-6 level, respectively). A positive regression between IL-6 and IL-1beta production by LPS stimulated monocytes was found both in patients ($r = 0.85$, $P < 0.0001$) and in controls ($r = 0.65$, $P = 0.001$) (Figure 3).

### Discussion

In this study we reported that cytokine production by monocytes is altered in patients with head and neck cancer in comparison to healthy subjects. An increased production of IL-6 by monocytes from cancer patients was observed in both unstimulated and LPS stimulated cells. Conversely, freshly isolated monocytes from both groups did not release detectable amounts of IL-1beta in the absence of stimulation and the increase in the release of this monokine after exposure to LPS was similar in cancer patients and controls.

The finding that monocytes from HNSC did not show any substantial differences in IL-1beta production when compared with IL-1beta release from controls, is in accordance with previous reports in patients affected by different neoplasms (Economou *et al.*, 1988; Arnould *et al.*, 1988; Erroi *et al.*, 1989). At variance are the results from other authors (Ikemoto *et al.*, 1990; Herman *et al.*, 1984; Pollack *et al.*, 1983) who found an altered capacity of monocytes to synthesise IL-1beta.

The increased IL-6 release by monocytes in HNSC patients could be related to a functional activation of monocytes in the host or to induction of this cytokine by other cytokines, such as IL-1beta and TNF-alpha. In fact, IL-1beta and TNF

**Figure 1** Monocyte IL-1 beta production in control subjects and in patients with head and neck cancer upon LPS stimulation (no statistically significant difference).

**Figure 2** Monocyte IL-6 production in control subjects and in patients with head and neck cancer upon LPS stimulation ($P < 0.05$).

**Figure 3** Relationship between IL-1beta and IL-6 monocyte production upon LPS stimulation in cancer patients and in control subjects.

may regulate IL-6 production in monocytes and in other cells (Tosato *et al.*, 1990; Kohase *et al.*, 1986). In our study IL-1beta has not been found higher in patients than in controls, but we should consider that IL-6, in its turn, inhibits IL-1beta production (Schindler *et al.*, 1990). Hence, a key role in the increased IL-6 production might be played by TNF-alpha, which induces appearance of IL-6 in peripheral blood when administered *in vivo* (Jablons *et al.*, 1988). Interestingly, we have previously observed an increased TNF-alpha production by peripheral blood monocytes in HNSC (Gallo *et al.*, 1991) possibly induced by tumour cell membrane costituents (Jaemcke *et al.*, 1990). In conclusion, our study demonstrated that the tumour bearing state may induce an altered cytokine release from peripheral blood monocytes characterised by an increased IL-6 production. Since IL-6 has multiple known biological effects including systemic control of hepatic acute phase protein synthesis, activation of T- B- and NK-cells, and effects on the adrenocorticoid axis (Hirano *et al.*, 1990), the high IL-6 release by monocytes may be one of the mechanisms underlying the host's immune and metabolic response to malignancy in these cancer patients.

### References

ARNOULD, R., GHANEM, G., DEBUSSCHERE, P., COMUNALE, J., LIBERT, A., VERCAMMEN-GRANDJEAN, A., EWALENKO, P. & LEJEUNE, F. (1988). Production of interleukin 1 by activated blood monocytes and alveolar macrophages from melanoma patients. *Anticancer Res.*, **8**, 775-780.

BEAUCHAMP, M.L. & WOLF, G.T. (1988). Monocytes and impaired leukocyte migration inhibitory factor production in head and neck squamous carcinoma. *Head & Neck Surg.*, Jan/Feb, 187-194.

BOYUM, A. (1976). Isolation of lymphocytes, granulocytes and macrophages. *Scand. J. Immunol.*, **5**, 9-15.

DINARELLO, C.A. (1988). Biology of IL-1. *FASEB J.*, **2**, 108-115.

ECONOMOU, J.S., COLQUHOUN, S.D., ANDERSON, T.M., McBRIDE, W.W., GOLUB, S., HOLMES, E.C. & MORTON, D.L. (1988). Interleukin-1 and TNF production by tumor associated mononuclear leukocytes and peripheral mononuclear leukocytes in cancer patients. *Int. J. Cancer*, **42**, 712-714.

ERROI, A., SIRONI, M., CHIAFFARINO, F., ZHEN-GUO, C., MENGOZZI, M. & MANTOVANI, A. (1989). IL-1 and IL-6 release by tumor-associated macrofages from human ovarian carcinoma. *Int. J. Cancer*, **44**, 795-801.

EVANS, R. & HASKILL, S. (1983). Activities of macrophages within and peripheral to the tumor mass. In *The Reticuloendothelial System*. A comprehensive treatise, Heberman, R.B. & Friedman, H. (eds), Vol. V, Cancer, pp. 155-176. Plenum: New York.

GALLO, O., GORI, A.M., ATTANASIO, M., MARTINI, F., FINI-STORCHI, O. & ABBATE, R. (1992). Interleukin-6 serum level and monocyte production in head and neck cancer. *Br. J. Cancer*, **65**, 479-480.

GALLO, O., PINTO, S., BOCCUZZI, S., DILAGHI, M., GALLINA, E., ATTANASIO, M., GORI, A.M., MARTINI, F. & ABBATE, R. (1991). Monocyte TNF production in head and neck squamous cell carcinoma. *Laryngoscope*, **102**, 447-450.

GAULDIE, J., RICHARDS, C., HARNISH, D., LANDSDORP, P. & BAUMANN, H. (1987). Interferon B2/BSF-2 shares identity with monocyte-derived hepatocyte stimulating factor 9HSF and regulates the major acute phase response in liver cells. *Proc. Natl Acad. Sci. USA*, **84**, 7251-7254.

HERMAN, J., KEW, M.C. & ROBSON, A.R. (1984). Defective interleukin 1 production by monocytes from patients with malignant disease. *Cancer Immunol. Immunother.*, **16**, 1852-1854.

HIRANO, T., AKIRA, S., TAGA, T. & KISHIMOTO, T. (1990). Biological and clinical aspects of interleukin 6. *Immunol. Today*, **11**, 443-449.

IKEMOTO, S., KISHIMOTO, T., IIMORI, H., MORIKAWA, Y., HAYAHARA, N. & MAEKAWA, M. (1990). Defective interleukin-1 production of monocytes in patients with bladder cancer. *Br. J. Urol.*, **65**, 181-185.

KOHASE, M., HENRIKSEN-DE STEFANO, D., MAY, L.T., VILCEK, J., SEHGAL, P. (1986). Induction of beta2-interferon by tumor necrosis factor: a homeostatic mechanism in the control of cell proliferation. *Cell*, **45**, 659-666.

JABLONS, D.M., MULE', J.J., McINTOSH, J.K., SEHGAL, P.B., MAY, L.T., HUANG, C.M., ROSEMBERG, S.A. & LOTZE, M.T. (1989). Induction by cytokine administration in humans. *J. Immunol.*, **142**, 1542-1547.

JAEMCKE, R. & MAENNEL, D.N. (1990). Distinct tumor cell membrane constituents activate human monocytes for tumor necrosis factor synthesis. *J. Immunol.*, **144**, 1144-1150.

MATSUSHIMA, K., ONOZAKI, K., BENZUR, M. & OPPENHEIM, J.J. (1987). Interleukin 1 as a potential tumoricidal factor. In *Physiologic, Metabolic and Immunologic Actions of IL-1*, Kluger, M., Oppenheim, J.J. & Powand, M.C. (eds) pp. Allan R. Liss Inc.: New York.

MORINGA, Y., SUZUKI, H., TAKATSUKI, F., ALKIYAMA, Y., TANIYAMA, T., MATSUSHIMA, K. & ONOZAKI, K. (1989). Contribution of IL-6 to the antiproliferative effect of IL-1 and TNF on tumor cell lines. *J. Immunol.*, **143**, 3538-3542.

MURAGUCHI, A., HIRANO, T., TANG, B. *et al.* (1985). The essential role of a B cell stimulatory factor 2(BSF-2/IL-6) for the terminal differentiation of B cells. *J. Exp. Med.*, **167**, 332-336.

POLLACK, S., MICALI, A., KINNE, D.W., ENKER, W.E., GELLER, N., OETGEN, H.F. & HOFFMANN, M.K. (1983). Endotoxin-induced in vitro release of interleukin-1 by cancer patient monocyte: relation to stage of disease. *Int. J. Cancer*, **32**, 733-736.

SCHINDLER, R., MANCILLA, J., ENDRES, S., GHORBANI, R., CLARK, S.C. & DINARELLO, C.A. (1990). Correlations and interactions in the production of interleukin-6, IL-1 and TNF in human blood mononuclear cells: IL-6 suppresses IL-1 and TNF. *Blood*, **75**, 40-47.

TAKAI, Y., WONG, G.G., CLARK, S.C., BURAKOFF, S.J. & HERRMANN, S.H. (1988). B cell stimulatory factor 2 is involved in the differentiation of cytotoxic T lymphocytes. *J. Immunol.*, **140,** 508–513.

TOSATO, G. & JONES, K.D. (1990). Interleukin-1 induces interleukin-6 production in peripheral blood monocytes. *Blood*, **75,** 1305–1310.

UICC. Union Internationale contre le Cancer (1978). TNM Classification of Malignant tumours. Third edition. Geneva: International Union against Cancer.

UNANUE, E.R. (1984). Antigen-presenting function of the macrophage. *Ann. Rev. Immunol.*, **2,** 395–401.

VAN DAMME, J., OPDENAKKER, G., SIMPSON, G.J., RUBIRA, M.R., CAYPHAS, S., VINK, A., BILLIAU, A. & VAN SNICK, J. (1987). Identification of the human 26 kd protein, interferon-beta 2, as a B-cell Hybridoma growth factor induced by IL-1 and TNF. *J. Exp. Med.*, **165,** 914–918.

WALTER, R.J. & DANIELSON, J.R. (1985). Defective monocyte chemotaxis in patients with epidermoid tumors of the head and neck. *Arch. Otolaryngol.*, **111,** 538–540.

WANEBO, H.J., RILEY, T., KATZ, D., PACE, R.C., JOHNS, M.E. & CANTRELL, R.W. (1988). Indomethacin sensitive suppressor-cell activity in head and neck cancer patients. The role of adherent mononuclear cell. *Cancer*, **61,** 462–474.